# Congenital toxoplasmosis and prenatal care state programs

**DOI:** 10.1186/1471-2334-14-33

**Published:** 2014-01-18

**Authors:** Mariza M Avelino, Waldemar N Amaral, Isolina MX Rodrigues, Alan R Rassi, Maria BF Gomes, Tatiane L Costa, Ana M Castro

**Affiliations:** 1 Pediatrics and Childcare Department of the Medical School of Federal University of Goiás (UFG), Goiânia, Brazil; 2 Department of Gynecology and Obstetrics of the Medical School of Federal University of Goiás (UFG), Goiânia, Brazil; 3 Clinical Analyses Laboratory, Clinical Hospital of UFG, Goiânia, Brazil; 4 Department of Ophthalmology of the Medical School (UFG), Goiânia, Brazil; 5 Maternal and Child Hospital of Goiania, Goiânia, Brazil; 6 Clinical Analyses Laboratory - Clinical Hospital of UFG, Goiânia, Brazil; 7 Laboratory studies of the host-parasite relationship at the Institute for Tropical Pathology and Public Health (IPTSP) of the Federal University of Goiás (UFG), - LAERPH/IPTSP/UFG, Goiânia, Brazil; 8 Department of Pediatrics and Puericulture MS/UFG and the Postgraduate Program from IPTSP/UFG, Rua 235 esq com 1a. Av. s/n Setor Leste Universitário, Goiânia-GO, Brazil

**Keywords:** Congenital toxoplasmosis, Pregnancy, Seronegative

## Abstract

**Background:**

Control programs have been executed in an attempt to reduce vertical transmission and the severity of congenital infection in regions with a high incidence of toxoplasmosis in pregnant women. We aimed to evaluate whether treatment of pregnant women with spiramycin associated with a lack of monitoring for toxoplasmosis seroconversion affects the prognosis of patients.

**Methods:**

We performed a prospective cohort study with 246 newborns (NB) at risk for congenital toxoplasmosis in Goiânia (Brazil) between October 2003 and October 2011. We analyzed the efficacy of maternal treatment with spiramycin.

**Results:**

A total of 40.7% (66/162) of the neonates were born seriously infected. Vertical transmission associated with reactivation during pregnancy occurred in 5.5% (9/162) of the NB, with one showing severe infection (systemic). The presence of specific immunoglobulins (fetal IgM and NB IgA) suggested the worst prognosis. Treatment of pregnant women by spiramycin resulted in reduced vertical transmission. When infected pregnant women did not undergo proper treatment, the risk of severe infection (neural-optical) in NB was significantly increased. Fetal IgM was associated with ocular impairment in 48.0% (12/25) of the fetuses and neonatal IgA-specific was related to the neuro-ophthalmologic and systemic forms of the disease. When acute toxoplasmosis was identified in the postpartum period, a lack of monitoring of seronegative pregnant women resulted in a higher risk of severe congenital infection.

**Conclusion:**

Treatment of pregnant women with spiramycin reduces the possibility of transmission of infection to the fetus. However, a lack of proper treatment is associated with the onset of the neural-optical form of congenital infection. Primary preventive measures should be increased for all pregnant women during the prenatal period and secondary prophylaxis through surveillance of seroconversion in seronegative pregnant woman should be introduced to reduce the severity of congenital infection in the environment.

## Background

Congenital toxoplasmosis adversely affects the eye, hearing, and brain function [1-16]. In Brazil, this fact was unknown until 2010, when mandatory reporting was implemented requiring the assessment of a program to control for congenital toxoplasmosis throughout this country [17]. Goiânia, the capital of Goiás, is located in the central-western region of Brazil. Goiânia has a high prevalence of toxoplasmosis in women of reproductive age (65.8%) [18]. Moreover, pregnant women in Goiânia have one of the highest serological conversion rates in the world (8.6%) [19], which represents a predisposition to congenital toxoplasmosis. This situation occurs because seronegative pregnant women undergoing immunological changes, which are typical of pregnancy [20], and those living in a location with a high prevalence of the disease, are more likely to acquire the infection [18,19]. This epidemiological risk stimulated the establishment of a state program to control congenital toxoplasmosis in October 2003. This program was linked to another pregnant woman care program created to help prevention of vertical transmission through primary and secondary prophylactic measures. These programs were created in an attempt to reduce vertical transmission and the severity of congenital infection.

Analyzing a screening programs’ effectiveness is essential for decision-making in public health politics. Seroprevalence data in pregnant women showed a decrease during the last 30 years in many European Countries, responsible for the discontinuation of some state programs control [21]. However, this has not happened in Brazil due to do the maintenance of the risk factors for acquiring this protozoal infection: low sanitation, feeding habits, contact with cats, contact with contamined soil, drinking beverages prepares unboiled water, consumption of municipal or uncontrolled water [18,19,22] and T. gondii virulence [15]. The infection during pregnancy is of concern for the consequences that may result in the fetus and this is of great. Even in an environment with a low incidence of infection, toxoplasmosis has proved to be important [23]. Countries that do not perform a prenatal control program for congenital toxoplasmosis have a higher frequency of severe forms of congenital infection [24-26]. Large European studies have questioned the effectiveness of preventive treatment of maternal infections in pregnancy [11,14,27-37]. Furthermore, prophylactic strategies against toxoplasmosis adopted by different public health systems are not always homogeneous [14,21,23,38-59]; they differ even within the same country. There is a high prevalence of toxoplasmosis in France where monitoring of seroconversion is performed monthly [14,45]. In most control programs toxoplasmosis in pregnancy surveillance seroconversion is held every three months (in three quarters), as in Austria [44] and Italy [50]. A program of prenatal screening was implemented in Slovenia [46] and Poland [53], countries with a low incidence of toxoplasmosis. And in other countries such as Denmark the screening program was discontinued [21]. In the United States [24] and United Kingdom [25,26], congenital toxoplasmosis is a rare condition. Therefore, these countries have not conducted any program for serological screening. There has been much discussion concerning the significance of these government programs to control toxoplasmosis in pregnancy [11,14,16,21,23,26,29,32-59]. Several facts hinder the enforcement of these protocols: identifying the acute phase of infection in pregnancy is difficult when seroconversion is not performed; proper interpretation of results of an IgG avidity test is difficult to achieve if it is carried out after 16 weeks of pregnancy [60-62]; treatment-compliance problems; inappropriate assistance to newborns (NB) according to the reference service; treatment provided for pregnant patients does not prevent vertical transmission of protozoan infection [11,14,16,27-40]; and the type of drug used to treat infected women causes international controversy [2,5-7,11,14,16,21,23,27-59].

Therefore, this study aimed to assess whether treatment of mothers for acute toxoplasmosis and a lack of surveillance of serological conversion in seronegative pregnant women affects their prognosis.

## Methods

### The population

We performed a prospective cohort study in 246 NB at risk of congenital toxoplasmosis, conducted between October 2003 and October 2011 at the Clinical Hospital (HC), Federal University of Goiás (UFG). This institution is a reference service for the control of congenital toxoplasmosis in Goiás, along with the Mother and Child Hospital of Goiania (HMI), and outpatient in Association of Parents and Friends of Exceptional Children (APAE). This study was conducted only with the children referred to the service reference HC/UFG. The HC is located in Goiânia. NB of women with specific IgM against T. gondii and low avidity IgG identified in the first serological examination prenatally and in NB of seronegative pregnant women who did not undergo monitoring for seroconversion during pregnancy were considered at risk for toxoplasmosis.

Mothers were selected by the Pregnancy Protection Program in the state of Goiás (PPGGO). These mothers were referred to the prenatal service at HC/UFG to continue the treatment initiated with spiramycin throughout pregnancy, once the presence of IgM in the peripheral blood of pregnant women, independent confirmation of fetal infection. But the HC/UFG also meets the demand of spontaneous pregnancy, which provided an opportunity surveillance of seroconversion in seronegative pregnant women seen at their prenatal service (low and high risk). HC in the seronegative pregnant women conducted surveillance of seroconversion for toxoplasmosis, repeat serologic testing in the second and third quarters. In the other services prenatal was not performed surveillance of seroconversion for toxoplasmosis among pregnant women at risk for toxoplasmosis. These patients were followed up until they gave birth at the maternity ward at HC/UFG. After birth, their NB were subjected to additional tests to determine the presence of congenital toxoplasmosis, according to the protocol 039/2002 approved by the Ethics Committee of the HC/UFG for Human and Animal Research.

NB were selected by: (1) routine postnatal screening in cord blood for detection of specific IgG and IgM antibodies against T. gondii; (2) postnatal screening (specific IgMs detected by the Guthrie test) in blood collected on filter paper from the digital pulp of NB between the 5th and 7th days of life - the sample was analyze by paper-based enzyme-linked immunosorbent assay (ELISA) with a sensitivity of approximately 100.0%, carried out at the Association of Parents and Friends of Exceptional Children (APAE) Goiânia and Anápolis; and (3) serological screening of peripheral blood of infants suspected congenital infection who were born at other public maternity hospitals that participate in the PPGGO. Routine postnatal screening was performed in NB who were born at the maternity ward of HC/UFG, and their mothers had specific IgM anti-T. gondii identified prenatally (by a screening test or serological monitoring). For serological screening, mothers were diagnosed with acute infection even though they had shown serological and/or clinical signs of congenital toxoplasmosis.

The detection of anti-T. gondii IgG and IgM in cord blood of NB suspected of congenital toxoplasmosis (mothers with specific IgMs or were seronegative who did not undergo surveillance of seroconversion prenatally) is routinely performed at the maternity ward of HC/UFG. The specific antibodies were confirmed in peripheral blood of NB and their mothers and were then compared. By seroconversion, this procedure identified women who had acute toxoplasmosis in pregnancy but were not diagnosed in the prenatal period.

### Medical and animal research ethics committee approval

The study was approved by the Human and Animal Experimentation Ethics Committee of the CH at the UFG (protocol no. 092/2001). Mothers of NB who agreed to participate signed a free informed term of consent after they have been made aware of the importance of the research. The protocol for diagnosis of congenital infection and treatment was approved by the Medical and Animal Research Ethics Committee from CH/UFG (039/2002).

#### Inclusion criteria

Vertical transmission diagnosis was confirmed in NB at risk of congenital toxoplasmosis whose mothers accepted to participate in the research. Exclusion criteria were as follows: (1) mothers who refused to take part in the research; and (2) inconclusive diagnosis of congenital infection, and NB whose mothers did not show seroconversion during pregnancy and who underwent neonatal screening (46 NB).

### PPGGO

This program is responsible for prenatal care for pregnant women attending public health services. The medical care in cases of prenatal low risk, is done in the Health Posts and Health Centers Assistance. In case of complications during pregnancy (risk pregnancies), medical care is performed in HC/UFG and Goiânia HMI. The pregnant women with acute toxoplasmosis during pregnancy are cared in three reference centers: HC/UFG, Goiânia HMI and ambulatory APAE. In addition, a serological survey of other infections that can be transmitted to the fetus was included in the PPGGO: syphilis, rubella, cytomegalovirus, human immunodeficiency virus (HIV), hepatitis B and C, human cell lymphotropic virus (HTLV), and Chagas disease at the time arrives to perform the pregnant the first prenatal consultation. Women with acute infection had a serological test for toxoplasmosis, which was performed on filter paper collected from blood from the digital pulp at the first time in the prenatal monitoring. Pregnant patients were considered to have an acute infection when a specific IgM was confirmed and the avidity of IgG (<30%) was low. Tests were performed before 16 weeks of pregnancy. These criteria were also used by Lapplainnen M et al. [60], Jenum et al. [61], and Werblin et al. [62]. Acutely infected patients were treated with spiramycin 3 g/day (from diagnosis to delivery), regardless of fetal infection or may not be present. When the patients started the prenatal monitoring after 16 weeks of pregnancy and the presence of specific IgM anti-T. gondii antibodies was confirmed, they underwent treatment because identifying the acute phase of protozoal infection was not possible. The diagnosis of fetal infection was carried out by identification of T. gondii by the following: (1) polymerase chain reaction (PCR) or isolation of T. gondii from mice; and (2) the presence of specific IgM antibodies in amniotic fluid and/or fetal blood from biological material collected after the 20th week of gestation by amniocentesis and cordocentesis, respectively. The procedures was performed by a specialist in fetal medicine.

### Congenital Toxoplasmosis Control Program (CTCP)

NB who met the criteria were included in the CTCP, which took place in the HC/UFG. In the HC/UFG, NB suspected of congenital infection were subjected to laboratory tests and routine procedures for the diagnosis of congenital infection, according to the service approved by the Ethics Committee of the CH/UFG (039/2002).

The CTCP considers a patient infected when: (1) T. gondii is isolated from peripheral blood or cerebrospinal fluid (CSF) by experimental inoculation in mice or DNA analysis with PCR; (2) specific anti-T. gondii IgM and/or IgA in fetal or NB blood is identified; (3) specific antibodies (IgG and/or IgM) are found in the CSF of NB; (4) the specific IgG (NB) is larger (4×) than maternal IgG; (5) NB IgG-specific antibody levels increase or remain positive after 12 months of life; and (6) a clinical alteration is compatible with the congenital infection in the absence of other diagnoses (Chagas disease, syphilis, rubella, cytomegalovirus, HIV, HTLV, and hepatitis B and C). The specific IgM and IgA were confirmed with a new blood sample collected between the 5th and 10th day of life. Fundus examination of the eye was performed at the Reference Center of Ophthalmology (CEROF), at the Department of Ophthalmology, Faculty of Medicine of UFG.

NB infected or suspected of being infected were treated according to the protocol approved by the Ethics Committee of the CH/UFG (039/2002). Treatment consisted of sulfadiazine (100–150 mg/kg/day) four times a day (every 6 hours), pyrimethamine (1–2 mg/kg/day) twice a day (every 12 hours), and folinic acid orally given in a daily dose of 3.5 mg. When protein levels in the CSF were >150 mg/dl or when there was acute ophthalmological impairment, a daily dose of prednisone (2–3 mg/kg/day) was added to the treatment. Drug doses were adapted to the neonatal period: sulfadiazine (100–150 mg/kg/day), twice daily in the first week of life; pyrimethamine (1–2 mg/kg/day) three times per week for 28 days. These conditions were maintained until there was certainty of no vertical transmission. However, for the infected patients, the duration of treatment depended on the presence of clinical abnormalities at birth (2 years) or on the absence of clinical signs (1 year).

### Laboratory techniques

#### Screening in pregnant women performed at the Institute of Diagnostic and Prevention (IDP) in the APAE in Goiânia

For the identification of specific anti-T. gondii in the blood of pregnant women, blood samples were collected by finger prick on filter paper S&S 903, according to standard procedures of the IDP in the APAE in Goiânia. Serological screening for toxoplasmosis in the eluate of filter paper was performed through ELISA kits, registered at the Ministry of Health by the National Agency for Sanitary Surveillance. The sensitivity of the filter paper technique for the identification of IgM anti-T. gondii was 99.8% [63] and the serum sensitivity was 97.9% (determined by the manufacturer). A similar study conducted in pregnant women from Mato Grosso do Sul state, in the mid-west of Brazil, found an IgM sensitivity in filter paper of 99.4% [64]. This test was repeated in the peripheral blood of pregnant women and confirmed by ELISA kits registered at the National Agency for Sanitary Surveillance. An IgG avidity test (before 16 weeks of gestation) was performed to determine if acute infection occurred before pregnancy (with high avidity). A low avidity test was considered as indicative of acute infection, as described in other studies [60-62].

Analysis of blood using the filter paper technique was described by Minuzzi [63]. Briefly, 3 μl of serum was used for the extraction of the IgM anti-T. gondii antibody, and this was placed in 200 μl of elution. The plate was homogenized at 1000 × g at room temperature for 1 hour and the tests were accomplished using an automated ELISA analyzer. The antibody capture method was based on ELISA. The presence of specific anti-IgM T. gondii allows the conjugated connect to the solid phase through the presence of toxoplasma antigen. The enzyme activity is proportional to the concentration of specific IgM present in the samples or controls. The enzymatic activity was analyzed by adding a colorless solution of chromogen/substrate, and was measured with a photometer. For measurement of enzymatic activity, 100 μl of calibrator and controls were added to the corresponding wells of a plate. Blood spots on filter paper (strip-sensitized) were eluted with 200 μl of the eluent. The plate was incubated for 60 minutes at 37°C and washed with washing buffer five times. A total of 100 μl of conjugate was dispersed in each well, and the plate was incubated for 60 minutes at 37°C. The plate was washed again with washing buffer five times. A volume of 100 μl of conjugate was placed in each well, and the plate was incubated for 30 minutes at room temperature protected from light. A volume of 100 μl of blocking solution was then added to each well and gently mixed for 30 seconds. The plate was then immediately read at 450 nm on a microplate reader.

#### Techniques used for the neonatal diagnosis of congenital toxoplasmosis

The following tests were performed at the Laboratory of Immunology HC/UFG (serology) and Laboratory Studies of the Host-Parasite Relationship (LAERPH) of the Institute of Tropical Pathology and Public Health (IPTSP) at UFG. These tests were used as markers of congenital infection with T. gondii. Serological tests to determine the presence of specific anti-T. gondii IgM and IgA antibodies were performed. Parasitological identification tests, such as experimental inoculation in mice and/or PCR to analyze biological samples suspected of being contaminated (fetal blood, amniotic fluid, blood, and CSF of NB) were also performed.

##### Parasitological examination

Blood samples were collected before specific medication was given to NB. These samples were taken to determine the presence of a parasite by PCR or experimental inoculation in mice (using according to Silva et al. [65]. PCR was performed according to the following protocol. DNA was extracted according to specifications from the Pure Link Genomic Purification kit for purification of genomic DNA (Invitrogen). The PCR reactions were performed in the MasterCycler personal thermocycler. The amplification process consisted of initial denaturation at 94°C (5 min), 35 cycles of denaturation at 94°C (1 min), annealing at 62°C (1 min), and extension at 72°C (1 min), followed by a final extension at 72°C (10 min). The PCR reactions were performed in duplicate, using a sequence of the B1 gene of T. gondii. The following primers were used: Toxo-B5 (5′-TGA AGA GAG GAA ACA GGT GGT CG-3′) and Toxo-B6 (5′-CCG CCT CCT TCG TCC GTC GTA-3′). The PCR products were visualized by 6% polyacrylamide gel electrophoresis and the gel was stained using silver nitrate [66]. Peritoneal fluid from mice infected with the T. gondii RH strain was used as a positive control.

##### Serological tests

Serological tests were used to detect anti-T. gondii IgG and IgM in blood samples of NB. Neonatal serological screening was performed using the indirect fluorescent antibody test (IFAT), according to Camargo et al. [67,68], and the microparticle enzyme immunoassay (MEIA). The IgM detection test for suspected patients of congenital toxoplasmosis was performed using the three techniques of IFAT, MEIA, and enzyme-linked fluorescent assay (ELFA). Methodologies used for detection of anti-T. gondii-specific antibodies were followed according to the manufacturer’s instructions. The specificity for IgM of the tests was 100.0%, except for IFAT-IgM, which was 91.7%. The sensitivity of the tests was 60.9% for MEIA-IgM, 60.9% for ELFA-IgM, 59.6% for IFAT-IgM, and 57.1% for ELISA-IgA (as described in Rodrigues IMX et al.), [69].

The IFAT technique was used according to Camargo et al. [67,68], with Biolab conjugate (Fluoline G and M). The presence of IgM was demonstrated by removal of rheumatoid factor, using reagents produced by Biomérieux. The reference values for IFAT were as follows: nonreactive, <1/10; and reactive, ≥1/10.

MEIA was used for the quantitative determination of anti-T. gondii IgG and IgM antibodies in the CSF (NB) or in the plasma of the pregnant women, NB, and puerperal patients. The MEIA was performed by following the instruction manual for the AXsYM ABBOTT immunochemical automated analyzer. Reference values for IgG were as follows: reactive, >3 UI/mL; indeterminate, 2–3 UI/mL; and nonreactive, <2 UI/mL. Reference values for IgM were as follows: reactive, >0.600 UI/mL; indeterminate, 0.500–0.600 UI/mL; and nonreactive, <0.500 UI/mL.

ELFA was used for the quantitative determination of anti-T. gondii IgM antibodies in the plasma of NB by following the instruction manual. Reference values for IgM using ELFA were as follows: nonreactive, <0.55 UI/mL; indeterminate, >0.55 and < 0.65 UI/mL; and reactive, >0.65 UI/mL (Biomérieux Instruction Manual Toxo-M). The ELFA was used because it involves immunocapture IgM antibodies. This process avoids false-positive results because of the presence of rheumatoid factor, and avoids false-negative results because of excess IgG, reactions. This procedure also has a sensitivity and specificity compared with ISAGA, of 93.5% and 99.3%, respectively.ELISA was used for the determination of IgA, according to the instruction manual. The specific anti-T. gondii IgA was examined using double-sandwich ELISA (capture). Reference values for specific IgA were as follows: nonreactive, <4.5 UA/mL; indeterminate, 4.5–5 UA/mL; and reactive, >5 UA/mL. Blood samples were sent to a private laboratory to detect anti-T. gondii IgM using the ELFA (VIDAS, Biomérieux) and IFAT [60].

### Statistical analysis

The data were computerized with Excel 2007 [SPSS 15.0 for Windows] was used for statistical analyses. We examined whether there was association between each of the variables. The results were compared with the presence and absence of maternal treatment with spiramycin. P values < 0.05 were considered as statistically significant with a 95% confidence interval. Fisher’s exact test was used when numbers were less than 5. Statistical calculations excluded cases where there was no information on the time of occurrence of the diagnosis of toxoplasmosis and maternal treatment.

## Results

The Protection Program for Pregnant women in Goiás made possible the diagnosis of 162 children infected with T. gondii (Table 1). These children were sent to the Reference Center in the HC/UFG.

**Table 1:** Distribution of clinical forms of congenital toxoplasmosis according to the type of maternal diagnosis of toxoplasmosis (2012, Goiânia/GO, Brazil)

**Clinical form**	**Maternal screening**			
	**IgM (+) %**	**Seroconversion %**	**Retired %**	**Total %**
With toxoplasmosis				55 (34)
1) Asymptomatic	30 (54.6)	14 (25.4)	11 (20.0)	34 (21)
2) Meningitis	26 (76.5)	8 (23.5)	0 (0.0)	11 (6.8)
3) Intracranial calcifications	8 (72.7)	3 (27.3)	0 (0.0)	38 (23.5)
4) Neural-optical	11 (28.9)	27 (71.1)	0 (0.0)	10 ( 6.2)
5) Ocular	6 (60.0)	4 (40.0)	0 (0.0)	7 (4.3)
6) Systemic	1 (14.3)	6 (85.7)	0 (0.0)	4 (2.4)
7) Auditory	4 (100.0)	0 (0.0)	0 (0.0)	3 (1.8)
8) Nodal	1 (33.3)	2 (66.7)	0 (0.0)	
**Sub-total**	87 (53.7)	64 (39.5)	11 (6.8)	162 (65.8)
**Without toxoplasmosis**	68 (80.9)	10 (11.9)	6 (7.2)	84 (34.2)
**Total**	155 (63.0)	74 (30.1)	17 (6.9)	246 (100.0)

Among the 246 women whose infants were followed up at the Reference Center for Congenital Infections, 30.1% (74/246) were seronegative at the first prenatal visit. Seroconversion was identified during gestation in 16.2% (12/74) of women who underwent prenatal care at the HC/UFG, which conducts monitoring of seroconversion in pregnant patients at risk of infection or seronegative women. Other women were diagnosed by the identification of specific anti-T. gondii IgG and/or IgM in the cord blood of NB. This was confirmed by the analysis of peripheral blood of NB and mothers.

The fetal IgM test was performed in 29.0% (47/162) of the cases and 53.2% (25/47) were positive. A total of 48.0% (12/25) cases where specific IgM was detected developed ophthalmological impairment. Neonatal IgM was positive in 38.5% (62/161) of the cases and neonatal IgA was positive in 20.4% (18/88). Patients with specific anti-T. gondii IgA (61.1% [11/18] developed a severe form of infection. A total of 85.7% (6/7) of the children with widespread disease were specific for IgA.

The parasite (T. gondii) was identified in 31.7% (38/120) of children and 34.2% (13/38) had a severe disability. In addition, CSF was abnormal in 70.9% (78/110) with T. gondii-specific IgG associated with a nonspecific condition. In 22 cases of congenital toxoplasmosis asymptomatic with abnormal CSF (28.2%), with no other laboratory markers of congenital infection, the change CSF helped in the diagnosis. Twelve of these cases had clinical signs late (6 children had chorioretinitis and 6 manifested seizures). In 13 NB IgG showed up in bonds much higher than maternal IgG. In the other 56 cases (71.8%) where changes were observed in the CSF, there was concomitant presence of other markers of congenital infection (IgM and/or IgA specific, and identification of T. gondii by PCR and/or mouse inoculation.

Among 162 infected patients, 40.7% (66/162) of them were born seriously sick. Among the seven patients who showed widespread disease, two also had neurological and optical impairments (Table 1). Furthermore, 30.8% (50/162) of the NB were born with ophthalmological impairment and 76.0% (38/50) of those also had neurological impairment. Among these, 20.0% (10/50) developed only the optical form, while 4.0% (2/50) had the systemic form of the disease. In addition, in these children, 52.0% (26/50) were born with poor eyesight, 50.0% (13/26) of these were blind, and 50.0% (13/26) had peripheral vision because of bilateral macular impairment. The systemic form was found in 4.3% (7/162), 31.5% (51/162) had intracranial calcifications, and among children with neurological damage, 37.2% (19/51) developed hydrocephalus.

There were no cases of systemic forms (Table 2), hearing or lymph nodes, in the third trimester of prenatal diagnosis. Among 62 seronegative pregnant women who were not monitored during pregnancy, and who had acute infection with T. gondii, 87.1% (54/62) had children infected and 64.8% (35/54) had severe forms of congenital infection (intracranial calcifications, neural-optical, ocular or systemic).

**Table 2:** Distribution of vertical transmission during the maternal diagnosis of toxoplasmosis (2012, Goiânia/GO, Brazil)

**Maternal screening**	**Without toxoplasmosis**		**Congenital toxoplasmosis**		**p**	**OR**
	**n**	**%**	**n**	**%**		
Before pregnancy	11	14.1	9	6.0		
1st trimester	35	44.9	43	28.5		
2nd trimester	18	23.1	24	15.9	< 0.001	1.694
3rd trimester	6	7.7	21	13.9		(1.349 – 2.128)
After birth	8	10.3	54	35.8		
Total	78	100.0	151	100.0		

Test: Kruskal Wallis.

Furthermore, 4.9% (8/162) of the patients were infected with toxoplasmosis and cytomegalovirus, and showed cerebral impairment characteristic of both infections.

Mortality associated with congenital infection was 4.3% (7/162).

A total of 45% (9/20) of women who had T. gondii infection before pregnancy (months before pregnancy or previous pregnancy) gave birth to infected children (Table 3). Four (44.4%) of those children developed an asymptomatic form of the disease, three (33.3%) had asymptomatic meningitis, one (11.1%) developed the systemic form of toxoplasmosis, and one (11.1%) had an ocular form of toxoplasmosis. Six cases of T. gondii infection occurred in previous pregnancies (1 neonatal death) and the other three occurred 3 months before pregnancy. No patient was infected by HIV or any other infectious disease (Chagas disease, syphilis, rubella, CMV, HTLV, and hepatitis B and C).

**Table 3:** Distribution of children by severe forms of congenital toxoplasmosis by time of gestation at which maternal infection was identified (2012, Goiânia/ GO, Brazil)

**Maternal screening**	**IC**		**Ocular**		**Neural-optical**		**Systemic**		**P**
	**N**	**%**	**N**	**%**	**N**	**%**	**N**	**%**	
Before pregnancy	1	9.1	1	10.0	―	0.0	1	14.3	
1st trimester	5	45.5	1	10.0	7	18.4	―	0.0	
2nd trimester	2	18.2	1	10.0	4	10.5	―	0.0	0.021
3rd trimester	1	9.1	3	30.0	4	10.5	―	0.0	
After birth	2	18.2	4	40.0	23	60.5	6	85.7	
Total	11	100.0	10	100.0	38	100.0	7	100.0	
P	0.297		0.406		0.001		0.001		

Test: x^2^.

IC = intracranial calcifications.

The use of spiramycin during pregnancy was conducted when the diagnosis of acute infection was established for presence of IgM antibodies in the bloodstream in pregnancy when associated with low avidity IgG (serological screening was conducted in the first prenatal visit); and when before pregnancy specific anti-T. gondii IgM persistence in the bloodstream, or with fetal compromise.) Furthermore, spiramycin was also used in the presence of seroconversion (specific anti-T. gondii IgG and IgM were identified in women who were previously seronegative). A total of 120 women were treated (Table 4). Among these women, 70 NB were born with congenital toxoplasmosis and 18.6% (13/70) of them were born severely infected. A total of 115 pregnant women were not treated. Among these women, 73% NB (84/115) had congenital infection and 60.7% (51/84) of them were born severely infected (Table 4). The neural-optical form of congenital infection occurred in all trimesters of pregnancy (Table 3). However, this form of infection (neural-optical) occurred in 36.9% (31/84) of NB of mothers who were not subject to antiparasitic treatment with spiramycin and it occurred in only 7.1% (5/70) when they were treated.

**Table 4:** Distribution of children by occurrence of congenital toxoplasmosis according to maternal treatment with spiramycin during pregnancy (2012, Goiânia/ GO, Brazil)

**Aspects**	**Maternal treatment**				**p**	**OR (IC)**
	**Treated**		**Untreated**			
	**N**	**%**	**N**	**%**		
1) Clinical type						
Mild form birth	57	81.4	33	39.3		0.148
Severe disease at birth	13	18.6	51	60.7	<0.001	(0.070-0.311)
Total	70	100.0	84	100.0		
2) Congenital toxoplasmosis						
Without toxoplasmosis	50	41.7	31	27.0	0.018	0.517
Congenital toxoplasmosis	70	58.3	84	73.0		(0.298-0.895)
Total	120	100.0	115	100.0		
3) Severe disease at birth						
Intracranial calcification	5	38.5	6	11.8		
Ocular	2	15.4	8	15.7		
Neuro-optical	5	38.5	31	60.8	0.030	0.377
Systemica	1	7.7	6	11.8		(0.185-0.765)
Total	13	100.0	51	100.0		
4) Mild form birth						
Assimptomatic	32	56.1	19	57.6		
Meningitis	20	35.1	12	36.4	0.321	1.222
Nodal	1	1.8	2	6.1		(0.678-2.201)
Auditory	4	7.0	0	0.0		
Total	57	100.0	33	100.0		

Test: x^2^.

Transmission of congenital toxoplasmosis was significantly associated with the time of diagnosis of maternal infection (p = 0,021), Table 3. Maternal diagnosis was associated with a significant difference as to severe forms of congenital infection (neuro-optical and systemic), in relation to the lack of diagnosis of maternal infection. The lack of diagnosis of maternal infection favored significantly congenital transmission of toxoplasmosis (Table 3).

41 pregnant women who had a diagnosis of toxoplasmosis during pregnancy did the indicated treatment. Of these, 26 had children infected and in 15 of them the children were not infected with T. gondii. Lack of treatment of pregnant women with spiramycin was significantly associated with the appearance of vertical transmission and a severe form of congenital infection (neural-optical) (Table 4). And the treatment of pregnant women decreased the incidence of severe fetal infection.

## Discussion

Although follow-up for serological conversion is recommended with a high prevalence of infection [12,14,23,42-47,60,70,71], this was not performed in the program state control of toxoplasmosis in Goiânia. Pregnancy increases the chances for infection to take place [72], especially in locations with a high prevalence of infection and major environmental contamination [22], contact with animal reservoirs, poor dietary habits, and low levels of formal education [18]. These conditions are experienced by pregnant women participating in prenatal services in public health throughout Brazil [18,19,22,72].

In our study, the diagnosis of congenital disease was difficult to establish [73,74]. Mothers suffered from acute toxoplasmosis or a recurrent form of the disease because of the low sensitivity of congenital infection markers. In NB, diagnostic markers of congenital toxoplasmosis showed a low sensitivity (IgM specific, 38.5%; IgA specific, 20.4%; and identification of T. gondii, 31.7%), smaller than that found by Bessieres et al. in 2009 [75], 64% (IgM) and 53% (IgA) Gilbert et al., [76], 52% (IgM) and 55% (IgA) and Pinon et al. [77], of 56.7%, in relation to serological markers. However, are similar to results obtained by Carvalheiro et al. [78] in Ribeirão Preto, Brazil. Also smaller to findings by Bessieres et al. [79] compared with identification of the parasite (61%). The identification of diagnostic markers of congenital toxoplasmosis differs depending on the treatment used during pregnancy. Couvreur et al. [71] identified the parasite in 42% of cases when used in association with pyrimethamine and sulfadiazine and 76% when using the spiramycin. In this study, IgM was also less identified among neonates whose mothers received sulfadiazine + pyrimethamine (17.4%) than among children of women who received spiramycin (69.2%). Bessières et al. in 2001 [79] also analyzed the effect of medication provided to pregnant women with acute toxoplasmosis according to the results of diagnostic markers of congenital infection. They also found reduction in the identification of the parasite in cord blood when the women were treated with pyrimethamine and sulfadiazine compared with little interference when spiramycin was used. Our findings of decreased transmission of severe forms of toxoplasmosis using only the spiramycin may be due to the fact that the strain of congenital toxoplasmosis in the Midwest region of Brazil (Goiânia) is more sensitive to spiramycin than the strain present in Europe.

These results imply that this type of infection should be further investigated, even though there is international debate on the subject that PCR performed on amniotic fluid is more important than other tests [80-85]. Foulon et al. [81] identified the T gondii in amniotic fluid in 81% of cases of toxoplasmosis; Bessieres et al. [79] identified of the parasite in placenta (60%) or umbilical cord blood (43%). Moroever, Knerer et al. [81], could not identify the importance of PCR in amniotic fluid.

Therefore, fetal-specific IgM proved to be a better marker for congenital infection than the peripheral blood NB test (53.2% and 38.5%, respectively). Found higher than those described by Foulon et al. [80] for specific IgM anti-T. gondii fetal (43%). However fetal-specific IgM can be considered a poor marker because it failed to be useful to diagnose nearly half of the infected patients. An important finding is that 48.0% of fetuses who presented with IgM in their bloodstream were born with ocular impairment. This finding has not been previously reported in the literature. In the current study, IgA-specific antibodies anti-T gondii were associated with the worst prognosis of vertical transmission (neural-optical and systemic forms), described also by Rodrigues et al. [69]. The specificity of tests was 100.0%, except for the IFAT-IgM (91.7%), but this was higher than that observed by Pinon et al. [77]. In other research, the meeting of specific IgA anti-T. gondii showed higher sensitivity, as Bessieres et al. (52%) [79] and Olariu et al. (43,9%) [24].

CSF is abnormal in 70.9% of infected patients and CSF is considered an important marker of congenital infection [5,7]. In our study, 28.2% (22/78) cases of congenital toxoplasmosis assyntomatical year born (with no other markers of congenital infection), changes in CSF helped in early diagnosis of congenital toxoplasmosis. Twelve of these cases had clinical signs late (6 had chorioretinitis and 6 manifested seizures). According to Alford [85] changes in CSF suggest neurological disease severity. However, Wallon et al. [86] found no significant association of CSF for the diagnosis of congenital toxoplasmosis in the neonatal period.

In our study, 5.5% (9/162) of congenital infection affected children of women immunocompetent who had acute infection prior to pregnancy. The majority of cases of vertical transmission in acute toxoplasmosis during pregnancy occur when maternal infection precedes gestation by several months [87-90]. In the current study, only three out of nine cases would fit that description, whereas the other six cases occurred in previous pregnancies. In addition, two children were born with severe infection. One of these children had the systemic form (his mother was diagnosed with acute toxoplasmosis earlier in pregnancy), and another child was born with the ocular form of the disease (the mother had acute toxoplasmosis 3 months before pregnancy). This finding suggests that this situation likely resulted from a reduced cellular response of the host during the gestational period [19]. This situation can interfere with the parasite load and with the clinical course of maternal infection, and consequently increase the risk of vertical transmission. Some authors have reported that IgG-positive pregnant women cannot transmit toxoplasmosis, except in rare cases of acute toxoplasmosis relapse due to immunodeficiency of the patient [5,7]. They showed that toxoplasmosis is a complex infection during pregnancy. Additionally, even the presence of prior immunity does not prevent the transmission of infection to the fetus. Furthermore, severe forms of vertical transmission were also found in these women in our study. This finding supports the establishment of preventive measures of a primary nature (in all pregnant women) to avoid contact with T. gondii by avoiding potentially contaminated food (meat, eggs, milk, and raw vegetables), contact with animals (cats and dogs), and land of gardens [4,5,7,37,58,91,92]. This because Elbez-Rubinstein [89] confirmed that acquired immunity against European Toxoplasma strains may not protect against reinfection by atypical strains.

Neurological manifestations, including asymptomatic meningitis, occurred in 52.5% (85/162) (Table 1), and 60% (51/85) of these children developed intracranial calcification, where 37.2% (19/51) had hydrocephalus. These results are also similar to those by Desmonts and Couvreur [2] in France and Safádi et al. (54%] in Brazil [93]. Furthermore, are larger than those found by Soares et al. [94] of 32%, also in Brazil. But much higher than that reported by Peyron et al. [13] of 11.8%; Foulon et al. [11], of 13% of sequels and Ricci et al. [36], of 31.6%. Our results that 40.7% (66/162) of NB were severely affected (Table 1) are better than those found by Olariu et al. [24], of 84% (when pregnant women and children were not treated). Gras et al. [16], showed that the treatment made the first 4 weeks of seroconversion reduced the risk of intracranial calcifications, but that was meaningless if started after that time, but did not affect the appearance of eye injuries.

Unidentified children at birth showed evolution of the congenital forms of the disease after birth, which is similar to results found in the classic studies of congenital infection [1,5,7]. Notably, these severe forms of congenital infections occurred more frequently when there was no diagnosis of acute infection in pregnant women. This finding can be explained by either a lack of serological surveillance of conversion in seronegative pregnant women or an absence of prenatal care. But in our state, we have a preventive program in pregnant women should have modified the severity of congenital infection. However, there were shortcomings in preventive measures both primary and secondary, which were associated with more aggressive strain of T. gondii circulating among us [15,95].

In the current study, among children with ocular impairment, 52.0% (26/50) were born with poor eyesight, and one half of them were blind and the other half had peripheral vision because of bilateral macular impairment. These results are in accordance with Peyron et al. [13], found that 58.8% of ocular lesions and 12.7% with low vision and a study by Gilbert et al. [15], who found more intense aggressiveness of toxoplasmosis in Brazil than in Europe. Moreover 4.3% (7/162) of children died as a result of severe congenital infection, similar to that found by Desmonts and Couvreur [2].

In our study, maternal treatment with spiramycin prevented the neural-optical form of the infection and a lack of treatment during pregnancy resulted in a greater risk for this alteration to occur (Table 4), results found in the different Gras et al. [16] without interference from the eye injury treatment. In Brazil, it has been observed that the severity of ocular toxoplasmosis than in other locations [15,95]. The findings of our study on the ocular are similar to that found by Peyron et al. [13], of 58.8%, but the severity of visual impairment was higher (52%) than the 12.7% found in the study of Peyron et al. [13], and lower than the 92.2% reported by Olariu et al. [24].

Congenital infection in general was also more severe because 4.3% (7/162) of children were born with the disseminated form of the disease, and two of these also had the neuro-optical form of the disease. The disseminated form of toxoplasmosis has been reported in other studies [96,97] and currently has been related to the strain II of T. gondii [96]. A high prevalence of the disseminated form was found in our study, which is considerably higher than that found in other countries. In studies carried out in Europe, complications, such as neonatal death and the systemic form, were reported, but the incidence did not exceed 1.0% of children [11]. Moreover, 4.9% (8/162) of children were infected by both toxoplasmosis and cytomegalovirus. Cerebral impairment is a characteristic of both infections and it made the prognosis of the infected patients even worse. These results are similar to previous studies classic congenital toxoplasmosis already published in the literature [1,2,5,7] and in locations that do not have a control state program for congenital toxoplasmosis [24,25]. The severity of clinical toxoplasmosis in Brazilian children may be associated with the genetic characteristics of T. gondii isolates prevailling in animals a humans in Brazil [22].

When patients were treated with spiramycin during pregnancy in our study, there was protection against this severe form of congenital infection and a decrease in frequency from 7.1% (6/84) to 1.4% (1/70). These results also suggest that there is a major flaw in the current toxoplasmosis control program of Goiás. The levels of severity of congenital infection need to be improved by implementation of primary prophylaxis in all pregnant women (seronegative and seropositive). Conduction of surveillance of seroconversion in pregnant women using serological tests performed on a monthly basis is also required to control congenital infection and identify maternal infection at an early stages of the infection. This would reduce transmission and the severe forms of congenital infection.

Hearing impairment was observed in 2.4% of infected children (Table 1). This finding was transient and disappeared with specific antiparasitic treatment in NB. Hearing changes were also found in other studies [5,98].

Our study found that a lack of monitoring of seroconversion in 62 pregnant women at risk during the 8 years of the PCTC in Goiania resulted in 56.4% (35/62) of children with severe forms of the infection (neurological, ocular and/or systemic). This number of children is much higher than that described by Desmonts and Couvreur [2] and Foulon et al. [11], and where there is no governmental program for controlling infection available [29,30]. This finding highlights the need for serological surveillance of pregnant women, as already previously recognized [5,7,14,37,50-59,91,92].

In our study, transmission of toxoplasmosis was higher when acute infection was identified postpartum and not during pregnancy. This is possibly related to the lack of maternal treatment. In surveys conducted in Europe, when acute infection occurred in the third trimester, no ocular or intracranial impairment was found in NB [51]. In contrast to our study results, no systemic, hearing, or lymph node impairment was found when the infection occurred in the third trimester of pregnancy. Moreover, neuro-optical forms found in all trimesters of pregnancy were present in 36.9% (31/84) of children when their mothers were not subjected to antiparasitic treatment with spiramycin during pregnancy. Only 7.1% (5/70) of children showed complications. Therefore, maternal treatment with spiramycin protected NB against neuro-optical impairment. A lack of treatment of acutely infected pregnant women also resulted in an increased risk of developing this type complication (Table 4). This severe form of congenital infection can manifest early at birth, as shown by the aggressive strain of T. gondii circulating in this study’s region of focus [15,96]. In addition, maternal treatment decreased the prevalence of these severe forms of toxoplasmosis from 60.7% (51/84) to 18.6% (13/70). These findings are much higher than those described by Schmidt et al. [21]. Previous studies have confirmed the importance of treatment of pregnant women with acute toxoplasmosis, even with spiramycin as a single drug [5,7,70]. Other studies favor the use of spiramycin in the first 16 weeks of pregnancy, followed by the association of pyrimethamine and sulfadiazine until delivery [14,27-36,39,42-47,50-59].

In our study, neurological and ocular aggressiveness, even in children of treated pregnant women, was greater than that found in areas lacking prenatal preventive programs. This indicates the need for future research, and isolation and genetic characterization of the circulating strains in Goiás, as well as the need for immune assays of neonates exposed to T. gondii in pregnancy.

In our study, although treatment of spiramycin was administered, there was clinical improvement of congenital infection consequences (sequelae). Moreover, Gras et al. [16] found no evidence that prenatal treatment with pyrimethamine-sulphadiazine was more effective than spiramycin in reducing the risks of intracranial or ocular lesions in congenitally infected infants.

However, these results with treatment are still higher than those found in the absence of treatment in other locations. Schimidt et al. [21] also found higher ocular aggressiveness among untreated women (9.6%) than in those who underwent treatment (2.8%).

Determination of specific anti-T. gondii IgM indicated in the new proposal of the Ministry of Health for prenatal programs in public health (Project Stork) is inadequate and ineffective based on the results of our study. This is because this proposal cannot identify seronegative pregnant women (or the risk of development of acute infection). This identification is important because the acute infection can only be confirmed as occurring during pregnancy when seroconversion is identified [5,7,19,72]. In addition to not performing primary prophylactic measures, which is effective for decreasing the rate of acute toxoplasmosis among susceptible women [5-7,44,46,56,58]. Moreover, treatment of the early infected fetus is delayed (secondary prevention). Our study highlights the need for preventive measures for all pregnant women, especially given that 5.5% of children who had congenital infection obtained the parasite from women immune to T. gondii and two of them developed severe forms of the infection (systemic and ocular).

Previous studies have shown that diagnosis of acute infection during pregnancy and its consequent treatment can reduce several forms of congenital infections [4-14,21,23,32,35,41-48,50-54,57-61],[80,81,86]. These studies emphasize the importance of preventive programs of congenital toxoplasmosis during pregnancy, including surveillance of seroconversion in seronegative pregnant women. In Brazil, where congenital infections are more aggressive than elsewhere in the world, a national program for controlling toxoplasmosis infection during pregnancy is highly recommended.

Our study has also indicated the need for improvement in the current program implemented in the state of Goiás. Primary and secondary prophylaxis measures should be intensified (e.g., serological surveillance in seronegative pregnant women and changes in the treatment of acutely infected patients). Monthly serological screening is suggested, such as the French program for control of congenital toxoplasmosis [14]. Notably, money spent on tertiary prophylaxis for severely infected NB and maintenance of life without quality exceeds what would be spent to carry out monthly serological tests in seronegative pregnant women (34.2%). According to Remington et al. [5], the expense is approximately one million dollars per patient with a severe form of congenital toxoplasmosis.

The treatment of pregnant women is a controversial issue. That’s because there are no controlled studies on the efficacy of the medication, but in absence of evidence of prenatal treatment effect does not exclude a clinically important beneficial effect. Our study is in accordance with studies that have shown a reduction in severity of fetal infection when the mother was treated during pregnancy [12,23,34-36,41-47,50-54,57-59]. Also showed that congenital toxoplasmosis is more severe in Goiânia than elsewhere [19]. Congenital toxoplasmosis occurs at much higher levels than in other locations that do not have a program of prenatal care. Implementation of primary preventive measures to control infection with T. gondii is recommended. These measures must be performed regardless of the patient’s immune status against toxoplasmosis to (1) implement monthly monitoring of seroconversion for seronegative pregnant women or for patients at risk, and (2) replace spiramycin by sulfadiazine after the 20th week of pregnancy (75 mg/kg/day in the first 2 days, followed by 50 mg/day in two doses) and give folinic acid (10–20 mg/day) until 1 week after withdrawal of drugs (up to the moment of birth). This treatment should be performed even without a diagnosis of fetal impairment because the diagnosis of congenital infections is complex and the aggressiveness of the infection in Brazil is severe. Notably, a positive test does not prevent vertical transmission.

## Conclusions

Treatment of pregnant women with spiramycin decreases the possibility of transmission of infection to the fetus. A lack of treatment is associated with the onset of the neuro-optical form of congenital infection. The occurrence of severe forms in pregnant women whose chronic infection is recurrent demonstrates the need to expand the primary prophylactic program to all pregnant women, regardless of their immune state against T. gondii. Occurrence of a severe form of congenital infection remains important in Brazil, despite government programs. The severity of congenital infection in Brazil suggests the requirement for secondary prophylaxis programs with drugs to treat the fetus and pregnant women who are acutely infected.

## Abbreviations

NB: Newborn; T. gondii: Toxoplasma gondii; PPGGO: Pregnancy Protection Program in the state of Goiás; HC: Clinical Hospital; UFG: Federal University of Goiás; APAE: Association of Parents and Friends of Exceptional Children; CTCP: Congenital Toxoplasmosis Control Program; CSF: Cerebrospinal fluid; PCR: Polymerase chain; HIV: Human immunodeficiency virus; HTLV: Human cell lymphotropic virus; CEROF: Reference Center of Ophthalmology; IDP: Institute of Diagnostic and Prevention in APAE; ANVISA: Ministry of Health by the National Agency for Sanitary Surveillance; LAERPH: Laboratory Studies of the Host-Parasite Relationship; IPTSP: Institute of Tropical Pathology and Public Health; IFAT: Indirect Fluorescent Antibody Test; MEIA: Microparticle Enzyme Immunoassay; ELFA: Enzyme-Linked Fluorescent Assay; ELISA: Enzyme-linked immunosorbent assay.

## Competing interests

All the authors declare that they have no competing interests.

## Authors’ contributions

All authors contributed to the design of the study, prepared and approved the final manuscript. MMA – Corresponding author, responsible for the toxoplasmosis project in Goiânia, the collection of results, data analysis, monitoring children suspected of congenital infection and writing the manuscript . WNA – responsible for collecting the biological material from the fetus and the mother with acute toxoplasmosis and for the treatment of the acutely infected pregnant woman; IMXR – responsible for conducting laboratory tests of the umbilical cord blood, NB suspected of congenital infection (beyond the 5th day of life) and cerebrospinal fluid, performed in the Laboratory of Immunology HC/UFG and also for laboratory monitoring of patients suspected of congenital infection; ARR - responsible for the ophthalmological testing of NB and to draft the manuscript; MBFG - partly responsible for of the monitoring of some NB and from writing study; TLC – carried out the immunoassays and participated in the writing of the manuscript. AMC – responsible for identification of Toxoplasma gondii by PCR and inoculacion in mice, participated in the project since its implementation and also responsible for the writing and analysis of the study data.

## Pre-publication history

The pre-publication history for this paper can be accessed here:

http://www.biomedcentral.com/1471-2334/14/33/prepub
